# Intermediate outcomes of ab externo circumferential trabeculotomy and canaloplasty in POAG patients with prior incisional glaucoma surgery

**DOI:** 10.1186/s12886-020-01645-0

**Published:** 2020-10-02

**Authors:** Huaizhou Wang, Chen Xin, Ying Han, Yan Shi, Sarah Ziaei, Ningli Wang

**Affiliations:** 1 Beijing Tongren Eye center, Beijing Tongren Hospital, Capital medical university, Beijing, China; 2 Department of Ophthalmology, University of California, San Francisco School of Medicine, San Francisco, California USA

**Keywords:** Ab externo circumferential trabeculotomy, Canaloplasty, Primary open angle glaucoma, Failed filtering glaucoma surgery

## Abstract

**Background:**

To compare the efficacy and safety of ab externo circumferential trabeculotomy (ECT) and canaloplasty on primary open angle glaucoma (POAG) patients with failed filtering surgery and intact schlemm’s canal (SC).

**Methods:**

We conducted a retrospective chart review of POAG patients with failed filtering surgery and intact SC, who further received ECT and canaloplasty. The primary outcome measures were intraocular pressure (IOP) and the number of topical medications at each follow-up point. The secondary outcome compared the quantified success rate at1-year follow-up between the groups.

**Results:**

Twenty-nine eyes were recruited in the ECT group and 19 eyes in canaloplasty group. The postoperative IOP and the number of topical medications decreased significantly in both groups (*p* < 0.001). The IOP at 3-month and 6-monthwas significantly lower in the ECT group (*p* = 0.039, *p* = 0.001) than in the canaloplasty group. Although the IOP at 12-mon was similar between the two groups, the number of topical medications was less in the ECT group (*p* = 0.040). Hyphema (72.4%) and ciliary body detachment (27.6%), which mainly resolve spontaneously, were two leading complications in the ECT group. The prevalence of hyphema was higher in ECT than in canaloplasty group (*p* < 0.001).

**Conclusion:**

For POAG with failed filtering surgery and intact SC, canaloplasty may be safer, whereas ECT presented better IOP control.

Translational Relevance: Suggestions for surgical choice for POAG with failed filtering surgery.

## Background

Glaucoma is the leading cause of irreversible blindness worldwide and it is estimated that 65.5 millions of population will involve in primary open angle glaucoma (POAG) in 2020 [1]. Surgical intervention is needed when patients fail in conservative treatment. Filtering surgery, such as trabeculectomy, is one commonly performed approach. The fistula between the anterior chamber and subconjunctival bleb is crucial for its success. However, the fistula is also involved in postoperative complications. Eventual formation of scar, blocking the fistula, leads to the failure of the surgery [2, 3]. It was reported that the failure rate of glaucoma filtering surgery reached 46.9% by postoperative 5-year follow-up and was even higher in patients with prior failed filtering surgery [4].

Bleb independent procedures, canaloplasty and ab externo circumferential trabeculotomy (ECT), were proposed this century and have become popular in clinic. It was reported that canaloplasty [5, 6] and ECT [7, 8] provide good IOP reduction with a favorable safety profile as an initial procedure for POAG. Moreover, canaloplasty is also a candidate for POAG with prior failed filtering surgeries with or without intact schlemm’s canal (SC) [9, 10]. Recently, a study reported that circumferential trabeculotomy could be a feasible remedy for POAG with failed incisional glaucoma surgery [11] and failed canaloplasty [12].

However, there were no studies that either directly compared the surgical outcome between canaloplasty and ECT or solely focused on the patient who had failed glaucoma filtering surgery. Here, we compared safety and efficacy of canaloplasty and ECT for POAG with failed glaucoma filtering surgery with 1-year follow-up.

## Methods

A retrospective chart review was performed for all POAG patients with failed filtering surgery who received canaloplasty or ECT procedures to further control IOP in the glaucoma department of Beijing Tongren Hospital between Feb 1st, 2014 and Dec 31st, 2016. All surgical procedures were done by senior glaucoma specialists, Dr. WHZ and WNL. The study followed the tenants of the Declaration of Helsinki and was approved by the institutional ethics committee at Beijing Tongren Hospital.

Inclusion criteria for this study were: (1) POAG patients received failed glaucoma filtering surgery, presenting with either an IOP > 21 mmHg or progressive deterioration of visual field defects on maximum tolerable medications. (2) Preoperative gonioscopic and ultrasound biomicroscopic examination revealed an open angle and identifiable intact Schlemm’s canal (SC). (3) No visible filtering bleb exists. Exclusion criteria were: other types of glaucoma, POAG with failed incisional glaucoma surgery and iris synechia or broken SC.

The full details of the surgical procedures were reported previously [9, 13]. Briefly, fornix-based conjunctival and scleral flaps were made next to the prior incision. Exposure of the lumen of SC was followed by the circumferential catheterization of SC. For canaloplasty, the viscoelastic (Healon GV, Abbott Vision, Abbott Park, IL, USA) was injected and 10–0 Polypropylene suture on tension was placed with the retraction of the microcatheter from the SC. If the circumferential catheterization could not be achieved, viscocanalostomy was done instead. For ECT, the two ends of the microcatheter were grasped and pulled in opposite directions. Thus, the trabecular meshwork (TM) was broken circumferentially. If the circumferential catheterization could not be achieved, TM was incised with Harms trabeculotome. Finally, the scleral and conjunctival flaps were sutured tightly in both groups.

After surgery, all subjects were given topical drops of combined tobramycin and dexamethasone for 2 weeks. The subjects receiving ECT were on topical 2% pilocarpine three times per day for 3 months.

The primary outcomes included IOP and the number of glaucoma medications at postoperative 1-month, 3-month, 6-month and 12-month follow-up visits. The secondary outcome was the success rate, which was defined as: (1) IOP ≤ 21 mmHg and a reduction in IOP ≥ 20% from baseline, (2) no additional glaucoma surgery was required, and (3) patients with or without medication.

All statistical analyses were performed with SPSS 18.0. Preoperative and postoperative measurements were compared using ANOVA analysis and Tukey paired comparisons. Independent t test was used to compare the IOP and medication between groups. Kaplan-Meier methods were used to calculate cumulative success rates for the two groups, which was compared with log-rank test. A *p* < 0.05 was considered statistically significant.

## Results

A total of 48 eyes in 45 patients were recruited, including 29 eyes (27 patients) in the ECT group and 19 eyes (18 patients) in the canaloplasty group. Demographics of the recruited patients are shown in Table 1. The types of prior glaucoma filtering surgery included trabeculectomy, Ex-PRESS shunt implantation and deep sclerectomy. The number of failed glaucoma surgeries was 1.3 ± 0.7 in the ECT group, including 6 eyes with 2 failed procedures and 1 eye with 3 failed procedures. The canaloplasty group had on average 1.3 ± 0.4 prior failed filtering surgeries, including 6 POAG eyes with 2 failed procedures. There is no significant difference between the two groups in either the number of prior surgeries (*p* = 0.787) or the interval between the last prior failed incisional glaucoma surgery and the bleb independent procedure (*p* = 0.394).

**Table 1:** Patient Demographics

Parameters	ECT	Canaloplasty	*P*-value
Number of eyes	29	19	
Sex			0.805
Male, n (%)	16 (65.5%)	10 (52.6%)	
Female, n (%)	11 (34.5%)	8 (47.3%)	
Side of eyes			1.00
Right, n (%)	15	9	
Left, n (%)	14	10	
Age, mean ± SD (year)	35.0 ± 7.1	40.1 ± 12.9	0.082
Number of prior surgeries, mean ± SD	1.3 ± 0.7	1.3 ± 0.5	0.787
Types of failed filtering surgeries	41	25	
Trabeculectomy, n (%)	35/41	18/25	0.214
Ex-PRESS shunt, n (%)	5/41	4/25	0.721
Non-penetrating, n (%)	1/41	3/25	0.148
Intervals mean ± SD (month)	19.8 ± 8.5	17.7 ± 8.0	0.394

*ECT* ab external circumferential trabeculotomy

Intervals: The interval between the last prior incisional glaucoma surgery and the bleb independent procedure

In the ECT group, 3 out of 29 eyes received TM incision with Harms trabeculotome. The postoperative IOPs were all significantly decreased from the baseline (*p* < 0.01). The IOPs at 6-mon and 12-mon were further decreased and significantly lower than IOPs at 1-mon (*p* = 0.029, *p* = 0.022). The number of topical medications was significantly decreased from baseline (*p* < 0.001), and it decreased further in 6-mon and 12-mon than that in 1-mon and 3-mon (*p* = 0.01, *p* < 0.01).

In the canaloplasty group, except two with viscocanalostomy, 17 eyes received circumferential catheterization and tensioning suture placement. The postoperative IOPs were all significantly decreased from the baseline (*p* < 0.01), but were not significantly different from each other. The number of topical medications was significantly different from the baseline (*p* < 0.01), but there was no significant difference since 1-mon and afterwards.

**Fig. 1:**
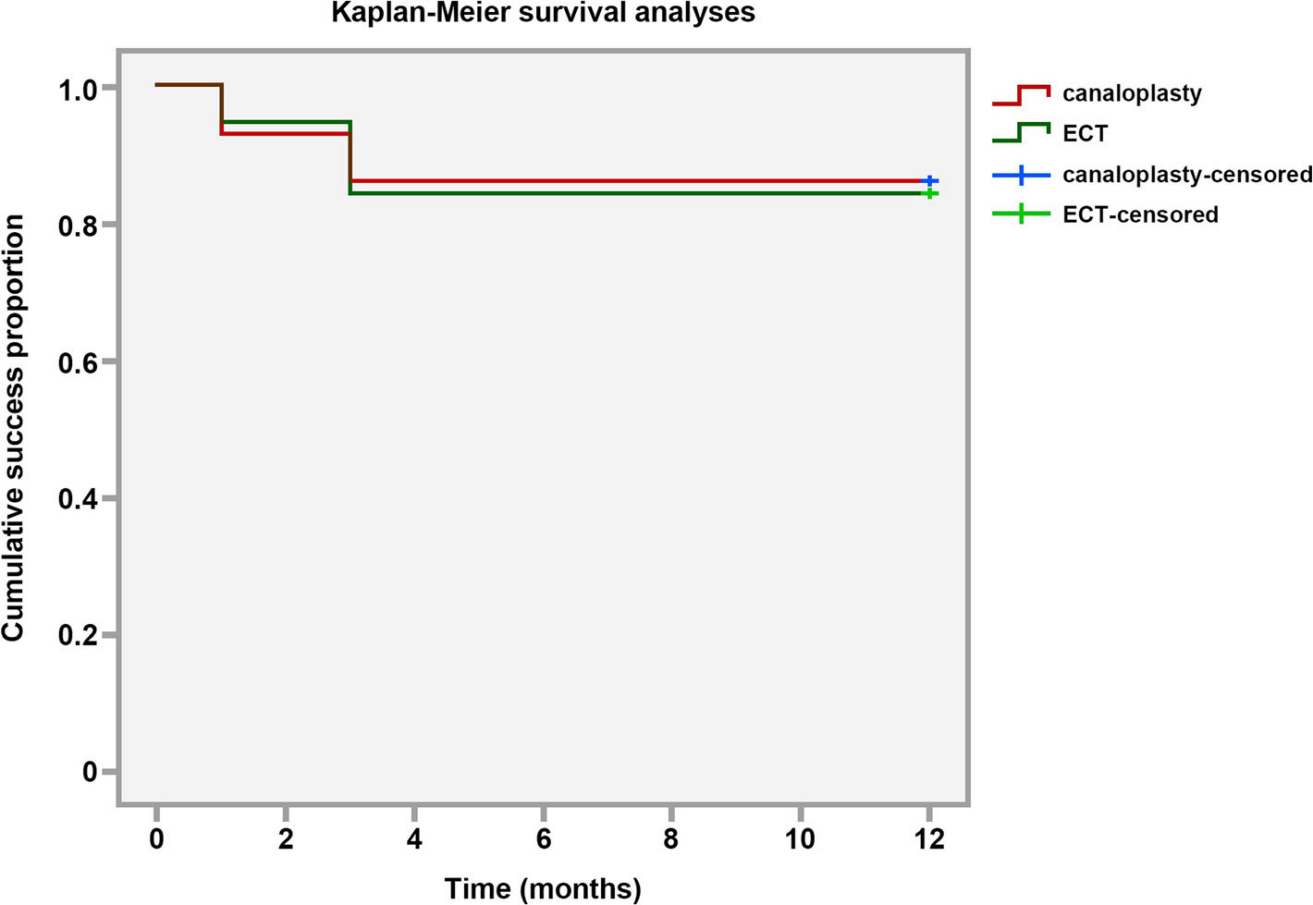
The cumulative probabilities of success of ECT and canaloplasty

In terms of comparison between groups, the postoperative IOPs at 3-mon and 6-mon were significantly lower in the ECT group than in the canaloplasty group (Table 2). The number of topical medications was similar between two groups at 1-mon, 3-mon and 6-mon postoperatively (*p* = 0.124, *p* = 0.668, and *p* = 0.053 respectively), whereas at 12-mon it was less in the ECT group than in the canaloplasty group (*p* = 0.040) (Table 3). The Kaplan-Meier survival analyses demonstrated that the one-year cumulative probabilities of success of ECT and canaloplasty was 86.2 and 84.2% respectively, which was not significantly different between groups (*p* = 0.866) (Fig. 1).

**Table 2:** Intraocular pressure (IOP) measurement (mmHg)

Parameters	ECT	Canaloplasty	*P*-value
Baseline	29.3 ± 9.6	29.5 ± 7.9	0.947
Postoperative (month)			
1-	19.0 ± 7.9	19.2 ± 5.4	0.896
3-	16.5 ± 3.5	18.6 ± 3.4	0.039
6-	15.2 ± 3.4	17.7 ± 1.2	0.001
12-	15.0 ± 2.5	16.3 ± 2.0	0.071
*P* value	< 0.001	< 0.001

**Table 3:** Number of topical medications (n)

Parameters	ECT	Canaloplasty	*P*-value
Baseline	3.2 ± 0.6	2.8 ± 0.9	0.089
Postoperative (month)			
1-	1.7 ± 2.0	0.9 ± 1.2	0.124
3-	1.0 ± 0.9	0.9 ± 1.2	0.668
6-	0.3 ± 0.7	0.9 ± 1.1	0.053
12-	0.3 ± 0.6	0.8 ± 1.0	0.040
*P* value	< 0.001	< 0.001

In the ECT group, all the patients presented intraoperative blood reflux into the anterior chamber. On day 1, all the patients demonstrated hyphema in different degrees, from scattered floating blood cells to 6 mm hyphema. The hyphema in 21 eyes (72.4%) resolved spontaneously in 7 days with no significant IOP increase. Among them,8 patients (27.6%) demonstrated ciliary detachment in anterior segment optical coherent tomography, which resolved spontaneously and did not lead to hypotony. Seven subjects experienced IOP spike during the postoperative 2 weeks. Among them were 6 patients on topical medication for whom IOP gradually decreased, and the medication was stopped within 2 weeks. Another patient received anterior chamber irrigation once. One patient presented hypotony and ciliary detachment for 7 days, and topical 2% pilocarpine was held until ciliary detachment resolved spontaneously.

In canaloplasty group, one subject presented hyphema with scattered blood cells (5.3%), which resolved spontaneously in 3 days. None experienced IOP spike within 2 weeks after surgery. One subject presented hypotony and ciliary detachment (5.3%) that resolved spontaneously in 1 week.

## Discussion

Repeated glaucoma filtering surgery in POAG patients leads to higher failure rate and complications. SC-based surgical procedures, devoid of bleb, presented good safety and efficacy and are probably of potential benefit to the POAG patient with failed glaucoma filtering surgery. Canaloplasty and ECT are two representative Ab externo procedures of SC-based glaucoma surgery. Canaloplasty was reported to be a feasible, efficient, and safe candidate for POAG with prior incisional glaucoma surgery with both intact and disrupted SC [9, 10]. Circumferential trabeculotomy, either ab interno or ab externo, was also practiced on POAG patients and presented good IOP control [7, 8]. Recently, a study reported that gonioscopy-assistant transluminal trabeculotomy appears to be safe and successful in treating 60 to 70% of POAG patients with prior incisional glaucoma surgery [11]. Meanwhile, some studies reported that the ab interno 360-degree suture trabeculotomy is a simple and safe technique to further enhance the IOP-lowering and drug-sparing effect of canaloplasty [14].

In this study, the circumferential catheterization of canal was achieved in 89.7 and 89.5% patients in ECT group and canaloplasty group. It was reported that besides the POAG-related changes in TM, continuous bypass of aqueous humor from the TM pathway further leads to more subendothelial deposits in TM and the amorphous material piling up in juxtacanulicular tissue. In turn, this leads to deterioration and collapse of the canal and decreased potential drainage ability [15, 16]. Unlike common blood vessels, the unique lymphatic-like phenotype of SC endothelium provides the potential lumen and recovery of the drainage even when it is collapsed for a long time, which remains the opportunity of the MIGS [17, 18].

Our study presented the similar 1-year cumulative quantified success rate of ECT and canaloplasty. In general, the postoperative IOP and the number of topical medications significantly decreased after either ECT or canaloplasty procedure. Although the subjects with ECT received pilocarpine continuously for 3 months in prevention of the scarring of the cut-edge along TM and the TM-iris adhesion, the POAG with ECT received a similar number of topical medications as canaloplasty at 1-mon, 3-mon and 6-mon. However, the postoperative IOP is lower in ECT at 3-mon and 6-mon than that in the canaloplasty group. At 12-mon, IOP were comparable for subjects with ECT and canaloplasty, but the number of topical medications in ECT group was lower than that in the canaloplasty group. These results implied that ECT may be relatively more efficient in IOP control than canaloplasty for the subjects recruited in this study, who had an average IOP of 30 mmHg with maximal topical medications. Previous study on normal cadaver human eyes showed that considering the initial IOP of 30 mmHg, the six-hour trabeculotomy or three-hour sinusotomy could proximately decrease 65% or 50% resistance of aqueous outflow [19]. In eyes with glaucoma, the resistance generated from TM dominated [20]. The ECT, probably eliminating all the resistance in the TM region, presents quick and good IOP control. Comparatively, canaloplasty reduces the IOP possibly by the mechanism of trans-descent window drainage, dilation of the SC and surrounding the collector channel (CC), recovery of the TM herniation in CC, disruption of the SC endothelium, and creation of more pores and cell junction detachment [21]. Thus, it directly decreased the resistance at the region of exposure of the SC and descent window, which is relatively less efficient than the circumferential trabeculotomy. However, it probably takes some time for regeneration of the drainage of the aqueous humor.

In terms of complications, canaloplasty seems to be safer. Canaloplasty did not induce any IOP spike or complication that needed to be resolved by further surgical procedure. Hyphema is more common and severer in the ECT group. Hypotony and ciliary body detachment is more frequent in the ECT group as well. Large fluctuation of IOP during the ECT procedure and repeated paracentesis could lead to ciliary body detachment [22]. Besides this, the potential for exacerbation of postoperative inflammation induced by the application of topical pilocarpine might be another reason for the ciliary body detachment and persistent hypotony [23]. Potential reasons for the IOP fluctuation in ECT patients include the recovery of the hypotony and ciliary body detachment and the aqueous drainage decrease in the uveaoscleral pathway induced by the topical pilocarpine [24].

Although it is proposed that topical pilocarpine could prevent the scarring of the TM cut-edge and the TM-iris adhesion, its potential effect on contraction of ciliary body and breakdown of the aqueous-blood barrier, which might increase failure of the ECT in the long run, warrant attention.

Repeated ab externo procedures and multiple scleral flaps may lead to substantial damage to the distal aqueous pathway, which is more severe in POAG with prior incisional surgeries. With newer surgical approaches, such as the gonioscopy assisted transluminal trabeculotomy (GATT), conjunctiva and scleral territory would be better preserved. This could lead to even better results for the repeat Schlemm’s canal surgery because of the preserved distal aqueous pathway.

The current study has several limitations. First, our sample size was small with one-year follow up. Second, the study was a retrospective study, although both groups were comparable. A randomized clinical trial should be performed to better understandthe efficacy of ECT and canaloplasty for POAG.

## Conclusion

In conclusion, for POAG with prior incisional glaucoma surgery, both canaloplasty and ECP decreased postoperative IOP and number of medications with high success rate at one-year follow up. While canaloplasty seems relatively safe with less postoperative complications, whereas ECT may achievebetter IOP control with fewer number of topical medications.

## Data Availability

The datasets used and analyzed during the current study are available from the corresponding author upon reasonable request.

## Abbreviations

ECT: Externo circumferential trabeculotomy
POAG: Primary open angle glaucoma
SC: Schlemm’s canal
IOP: Intraocular pressure
TM: Trabecular meshwork
CC: Collector channel
GATT: Gonioscopy assisted transluminal trabeculotomy
